# Effect of Storage Temperature on Cultured Epidermal Cell Sheets Stored in Xenobiotic-Free Medium

**DOI:** 10.1371/journal.pone.0105808

**Published:** 2014-08-29

**Authors:** Catherine Jackson, Peder Aabel, Jon R. Eidet, Edward B. Messelt, Torstein Lyberg, Magnus von Unge, Tor P. Utheim

**Affiliations:** 1 Department of Medical Biochemistry, Oslo University Hospital, Oslo, Norway; 2 University of Oslo, Oslo, Norway; 3 Ear, Nose and Throat Department, Division of Surgery, Akershus University Hospital, Lørenskog, Norway; 4 Institute of Clinical Medicine, University of Oslo, Oslo, Norway; 5 Department of Oral Biology, Faculty of Dentistry, University of Oslo, Oslo, Norway; 6 Centre for Clinical Research, LT Vastmanland, Uppsala University, Uppsala, Sweden; University of Newcastle upon Tyne, United Kingdom

## Abstract

Cultured epidermal cell sheets (CECS) are used in regenerative medicine in patients with burns, and have potential to treat limbal stem cell deficiency (LSCD), as demonstrated in animal models. Despite widespread use, short-term storage options for CECS are limited. Advantages of storage include: flexibility in scheduling surgery, reserve sheets for repeat operations, more opportunity for quality control, and improved transportation to allow wider distribution. Studies on storage of CECS have thus far focused on cryopreservation, whereas refrigeration is a convenient method commonly used for whole skin graft storage in burns clinics. It has been shown that preservation of viable cells using these methods is variable. This study evaluated the effect of different temperatures spanning 4°C to 37°C, on the cell viability, morphology, proliferation and metabolic status of CECS stored over a two week period in a xenobiotic–free system. Compared to non-stored control, best cell viability was obtained at 24°C (95.2±9.9%); reduced cell viability, at approximately 60%, was demonstrated at several of the temperatures (12°C, 28°C, 32°C and 37°C). Metabolic activity was significantly higher between 24°C and 37°C, where glucose, lactate, lactate/glucose ratios, and oxygen tension indicated increased activation of the glycolytic pathway under aerobic conditions. Preservation of morphology as shown by phase contrast and scanning electron micrographs was best at 12°C and 16°C. PCNA immunocytochemistry indicated that only 12°C and 20°C allowed maintenance of proliferative function at a similar level to non-stored control. In conclusion, results indicate that 12°C and 24°C merit further investigation as the prospective optimum temperature for short-term storage of cultured epidermal cell sheets.

## Introduction

Preparation of cultured epithelial cell sheets (CECS) for clinical use requires a high level of expertise and specialized facilities. Tissue generation laboratories are subject to high safety and quality standards [1]. These conditions represent a barrier to the widespread use of CECS while demand is anticipated to increase as a result of research and clinical success [2]. Development of a reliable storage option for cultured cells would enable wider distribution from centralized laboratories to clinics worldwide [3]. In addition, a storage interval provides increased opportunity for quality control [4]. Current methods employed in the storage of epidermal cells include refrigeration of whole skin grafts and cryopreservation of cultured epithelial cell sheets (CECS). Poor viability (reduction to 50% within three days of storage), has been shown following refrigeration (4°C) of whole skin grafts in saline, which is the most common method of storage used in burns units according to a recent survey [5]. While some cryopreservation studies have shown relatively good cell viability [6], there are several examples of disintegration and unusable CECS architecture [7], as well as low cell viability using this method [8]–[10]. Moreover, it has been shown that cryopreserved skin must be used within two days upon thawing, as cell viability rapidly diminishes [11]. These disadvantages, coupled with the need for complicated freeze/thaw schedules and specialized equipment, makes reliable storage of CECS at above-freezing temperatures a promising alternative.

The treatment of large area burns and limbal stem cell deficiency (LSCD) are two applications that would especially benefit from the development of short-term storage by providing improved access and an extended interval for quality control. In the treatment of burns, a small biopsy taken from intact skin can be expanded to produce enough CECS to cover an adult body within three or four weeks [2]. Use of CECS is especially suitable when extensive injury does not allow the use of split-skin grafts. A reliable and convenient storage option would aid in flexible scheduling of surgery with respect to patient readiness, and provide reserve sheets for repeat operations within a certain interval, benefits that are particularly relevant to burns units when working with unstable patients [12].

LSCD is a painful disease caused by loss or damage to stem cells located at the periphery of the cornea, the limbus. Defects in the corneal epithelium and loss of vision may significantly reduce quality of life [13]. In 1997 Pellegrini *et al*. demonstrated that a small limbal biopsy taken from the patients' contralateral healthy eye could be cultured *in vitro* to provide an epithelial cell sheet for treatment of LSCD [14]. Almost 1000 cases of treatment using CECS have since been documented, with an overall success rate of approximately 75% [15].

With the goals of minimizing risk of damage to the healthy, or less damaged eye, and reducing exposure to immunosuppressive drugs, the use of an alternative autologous cell source holds great potential. Initial animal and human studies using alternative cell types have shown promising results. Examples include the use of cultured epidermal keratinocytes to treat damaged cornea in goat [15]–[17]. To date only cultured oral mucosa cells [18], cultured conjunctiva cells [19], and limbal cells [20] have been used successfully in the treatment of LSCD in humans. However, the abundance, ease of availability, low risk associated with harvest, and large expansion potential suggests that keratinocyte stem cells derived from a patient skin biopsy may be an ideal source of autologous cells in the treatment of LSCD [21].

The overall objective is therefore to establish optimal storage conditions for CECS to facilitate improved transplantation success and extend access to regenerative medicine. The purpose of this study was to first assess the effect of a range of above-freezing temperatures as an initial step towards achieving this objective.

## Materials and Methods

### Supplies

Normal adult human epithelial keratinocytes (HEKa) and Keratinocyte Medium (KM) were obtained from ScienCell Research Laboratories (San Diego, CA). The provided cells originated from an 18 year old female undergoing breast-reduction surgery. Goat serum, trypsin-ethylenediaminetetraacetic acid (EDTA), 4-(2-hydroxyethyl)-1-piperazineethanesulfonic acid (HEPES), sodium bicarbonate, sodium azide, Tween-20, Triton X-100, gentamycin, bovine serum albumin (BSA), fetal bovine serum (FBS), 4', 6-diamidino-2-phenylindole (DAPI), were purchased from Sigma Aldrich (St Louis, MO). Nunclon Δ surface multidishes, glass coverslips, pipettes and other routine plastics were obtained from Thermo Fisher Scientific (Waltham, MA). Phosphate buffered saline (PBS) and minimum essential medium (MEM) were from Life Technologies (Carlsbad, CA).

### Cell Culture

HEKa were seeded (5000 cells/cm^2^) in serum-free KM (0.09 mM Calcium) on Nunclon Δ surface multidishes or glass coverslips. Cells were cultured in a 5% CO_2_ incubator at 37°C for 5–7 days to obtain a confluent (90–100%) monolayer. Culture medium was changed every two days. To minimize confounding variation and to focus on temperature-associated differences, cells came from a single donor and were cultured on uncoated plastic, thereby limiting variation in the form of a biological substrate (e.g. amniotic membrane or fibrin).

### Cell Storage

Following culture, each multidish was sealed and randomly selected for storage at one of nine different temperatures 4°C, 8°C, 12°C, 16°C, 20°C, 24°C, 28°C, 32°C and 37°C (n = 4 for each temperature). The standard deviation of the temperature in each storage container was ±0.4°C as demonstrated previously [22]. The storage medium consisted of MEM with 12.5 mM HEPES, 3.57 mM sodium bicarbonate and 50 *µ*g/ml gentamycin. Cultured HEKa, not subjected to storage, served as controls. Cells were stored for 14 days in tightly sealed air-tight culture wells by means of Nunclon adhesive sheets. Following storage, MEM storage medium was replaced with KM, and cells were allowed to equilibrate in the 37°C incubator for 3 hours before all analyses in order to assess any potential damage incurred upon rewarming [23]. An extended storage period of 14 days was chosen to accentuate any temperature associated differences.

### Assessment of Cell Viability

HEKa cells (n = 4) were incubated with calcein AM (CAM) (1∶2000) which permeates the cell membrane and is hydrolyzed by live cells to yield green fluorescence, and ethidium homodimer-1(EthD-1) (1∶1000) which permeates the membrane of dead cells, and labels nucleic acids to yield red fluorescence (Invitrogen Live/Dead Analysis Kit, Life Technologies, Grand Island, USA). Incubation was at room temperature for 45 minutes. Fluorescence was measured with a microplate fluorometer (Fluoroskan Ascent, Thermo Scientific, Waltham, MA) with the excitation/emission filter pairs 485/538 for CAM and 530/620 for EthD-1. Background fluorescence, measured in wells containing CAM and EthD-1 without cells, was subtracted from all values.

### Metabolic Analysis

Metabolic readings taken from the storage medium were obtained directly upon removal of stored cells from storage at the nine different temperatures. Oxygen tension (pO_2_), glucose, lactate, and pH values were measured using a Radiometer ABL 700 blood gas machine (Bronshoj, Denmark). The analyzer was automatically calibrated following the manufacturer's protocol prior to analysis (Radiometer ABL 700 User Manual).

### Light Microscopy

Light microscopy images were taken at 400X magnification, using a Leica DM IL LED microscope and Canon EOS 5D mark II camera (Canon, Oslo, Norway). Images were processed using Photoshop version CS6 extended software.

### Scanning Electron Microscopy

HEKa cells cultured on glass coverslips were prepared for scanning electron microscopy (SEM) as previously described [24]. In brief, glutaraldehyde-fixed samples (n = 3) were dehydrated in increasing ethanol concentrations, then dried according to the critical point method (Polaron E3100 Critical Point Drier; Polaron Equipment Ltd., Watford, UK) with CO_2_ as the transitional fluid. The specimens were attached to carbon stubs and coated with a 30 nm thick layer of platinum in a Polaron E5100 sputter coater before being photographed with an XL30 ESEM electron microscope (Philips, Amsterdam).

### Immunocytochemistry

Cells cultured in Nunclon 24-well multidishes (n = 4) were rinsed in PBS, fixed in ice-cold 100% methanol, incubated at −20°C for 7 minutes and washed with fresh PBS. Fixed cells were incubated in a 10% goat serum blocking buffer - 10% goat serum, 1% BSA, 0.1% Triton X-100, 0.05% Tween-20, 0.05% sodium azide in PBS, for 45 minutes at room temperature with gentle shaking. Cells were then incubated overnight at 4°C or at room temperature for 1 hour in a humidified chamber, with primary antibody to Proliferating Cell Nuclear Antigen (PCNA) (DAKO, Glostrup, Denmark) diluted in the same 10% goat serum blocking buffer. CY3-conjugated secondary antibodies were diluted in 0.2% PBS-Tween 20 with 1% BSA and incubated for one hour at room temperature. Addition of PBS in place of the primary antibody served as a negative control. The cells were rinsed three times in PBS before a wash containing 1 *µg*/mL DAPI to stain cell nuclei followed by a final wash with PBS. Using a magnification of ×200, random positions were selected for image capture using an inverted epi-fluorescence microscope (Nikon Eclipse Ti with a DS-Qi1 camera; Nikon Instruments, Tokyo, Japan). The exposure length and gain were kept constant. ImageJ software was used to process the images. The percentage and standard deviation of positive staining was calculated based on an average calculated from counting ∼100 cells from randomly selected positions in 4 wells.

### Statistical Analysis

One-way ANOVA with Tukey's post hoc pair-wise comparisons (SPSS ver. 19.0) was used to compare the groups. Correlations were made using Pearson correlation. Data were expressed as mean ± standard deviation, and values were considered significant if *p*<0.05.

## Results

### Cell Viability and Cell Death Values

**Figure 1:**
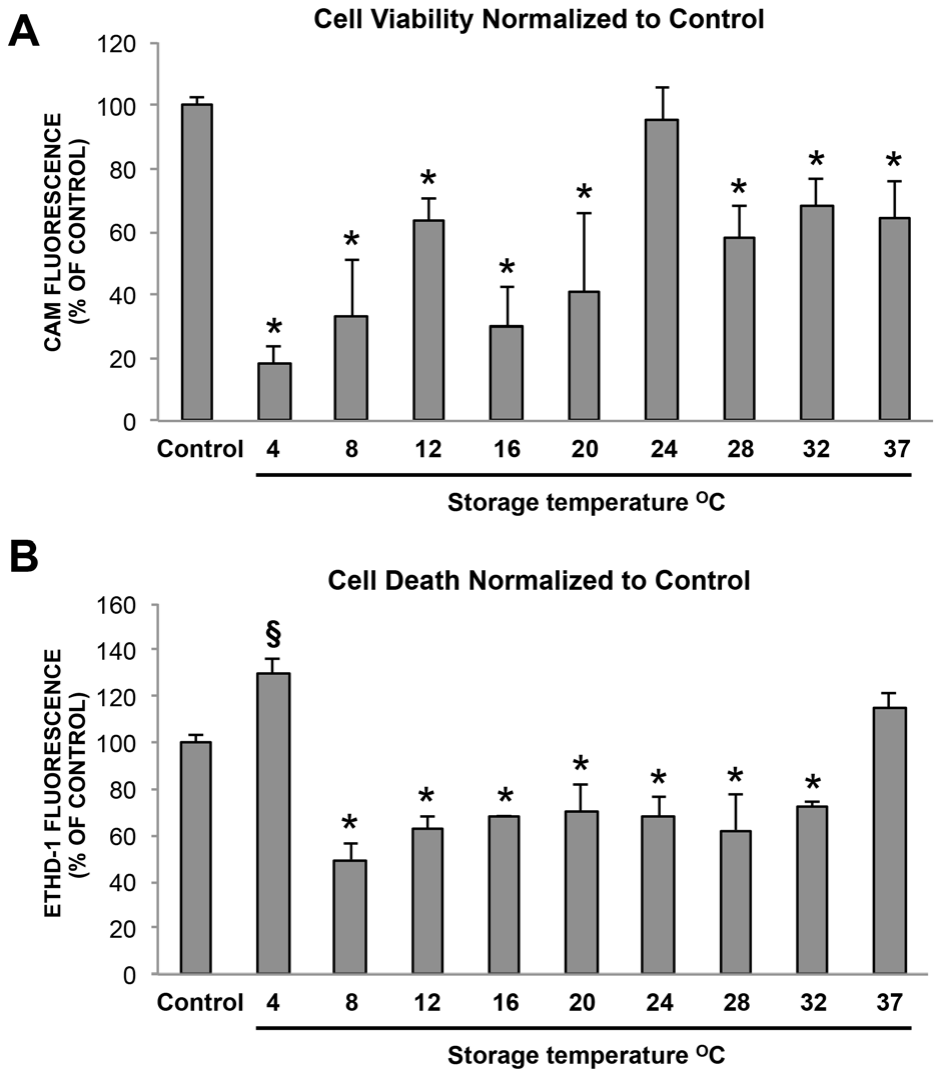
Cell viability and cell death after two weeks storage. (A) Cell viability (CAM fluorescence). Cell viability was clearly best maintained at 24°C with a value of 95.2 ± 9.91% compared to non-stored control cells (*p*  =  0.984). All other temperatures had significantly reduced cell viability compared to non-stored control cells (*p* < 0.001). (B) Dead (EthD-1 fluorescence) values (n = 4). *  =  significantly lower compared to control (*p* < 0.05) §  =  significantly higher than control, (*p* < 0.001) (n  =  4).

**Figure 2:**
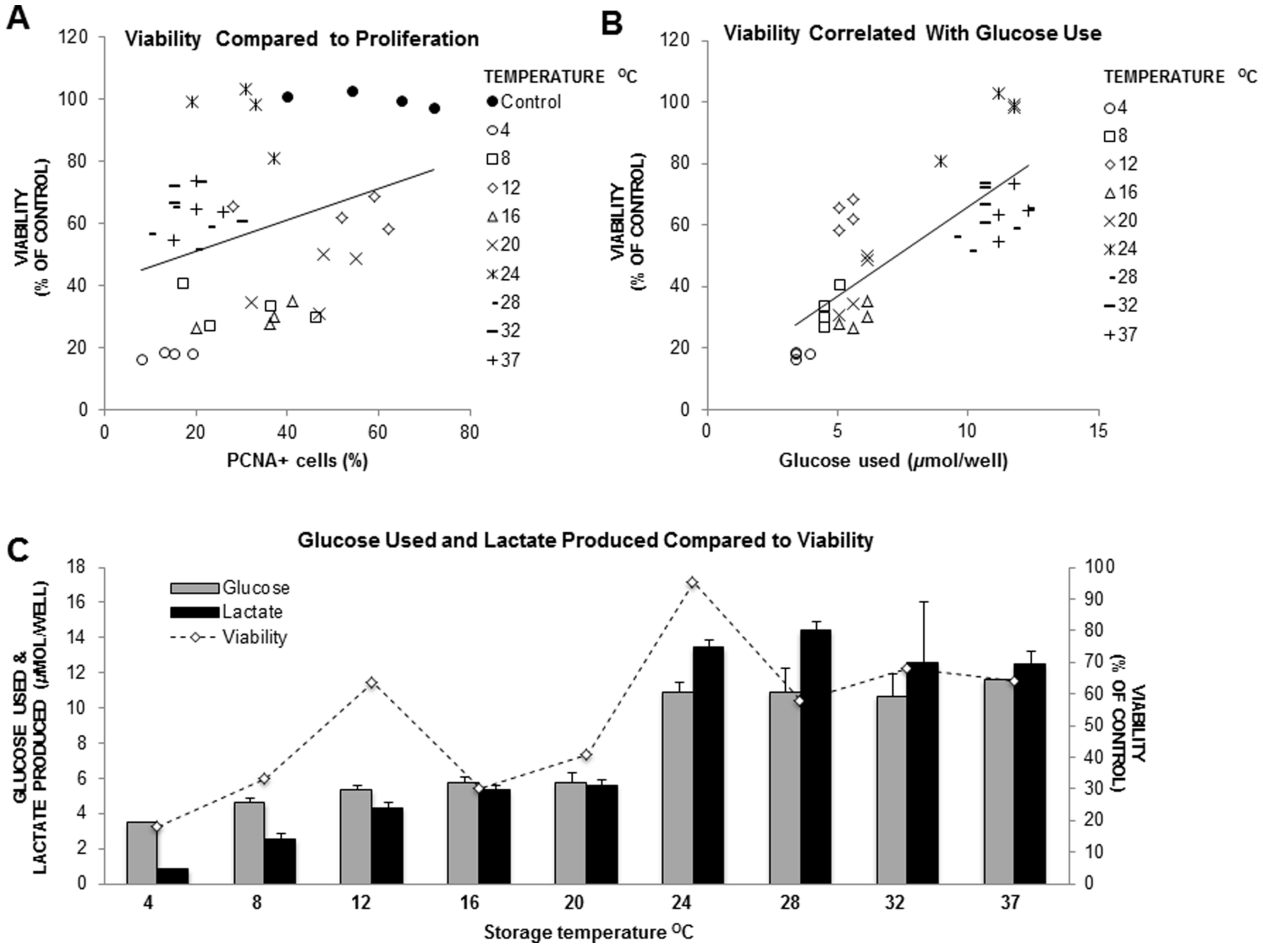
Cell viability comparison with PCNA, glucose use, and lactate production. (A) Correlation between cell viability and PCNA expression shows a distinct separation between high and low temperatures within the dataset (*r*  =  0.316; *p* < 0.05). (B) Cell viability was also correlated with glucose use (*r*  =  0.782; *p* < 0.001). (C) Cell viability compared to metabolic values by temperature. Values represent the average seen in n  =  4 wells of a 12 well plate at each temperature.

Calcein (CAM)/Ethidium Homodimer-1 (EthD-1) values reflect the relative live/dead numbers within the cell population remaining after removal of detached dead cells. Cell viability was clearly best maintained at 24°C with a value of 95.2±9.9% compared to non-stored control cells (*p* = 0.984) (Figure 1A). All other temperatures had significantly reduced cell viability compared to non-stored control cells (*p*<0.001). However, ∼60% cell viability was conserved at 12°C, 28°C, 32°C, and 37°C. Cell viability was significantly correlated with PCNA expression (*r* = 0.316; *p*<0.05) (Figure 2A), and with glucose use (*r* = 0.782; *p*<0.001) (Figures 2B and 2C), throughout the temperature range.

Significantly higher cell death compared to the non-stored control was seen at 4°C (*p*<0.001) and there were also more dead cells at 37°C (Figure 1B). Cell death was significantly lower at all temperatures between 8°C and 32°C where values fluctuated at ∼60% of the non-stored control cells (*p*<0.001 for all the groups). This may reflect detachment and removal of floating dead cells during storage and processing.

### Metabolic Status

The metabolic status of cells stored in three different volumes (24-well, 12-well and 6-well plates) was assessed by measurements of glucose, lactate, pO_2_ and pH in the media at the end of the storage period. The metabolic values taken from the cell viability experiment were investigated separately for correlation with cell viability results. Media without cells served as a control. The average glucose and lactate concentration in KM (used in cell culture before storage) was 5.04±0.32 mMol/L and 1.47±0.44 mMol/L respectively.

### Glucose Use, Lactate Production, Oxygen Tension, and pH in the Cell Viability Experiment Group

**Figure 3:**
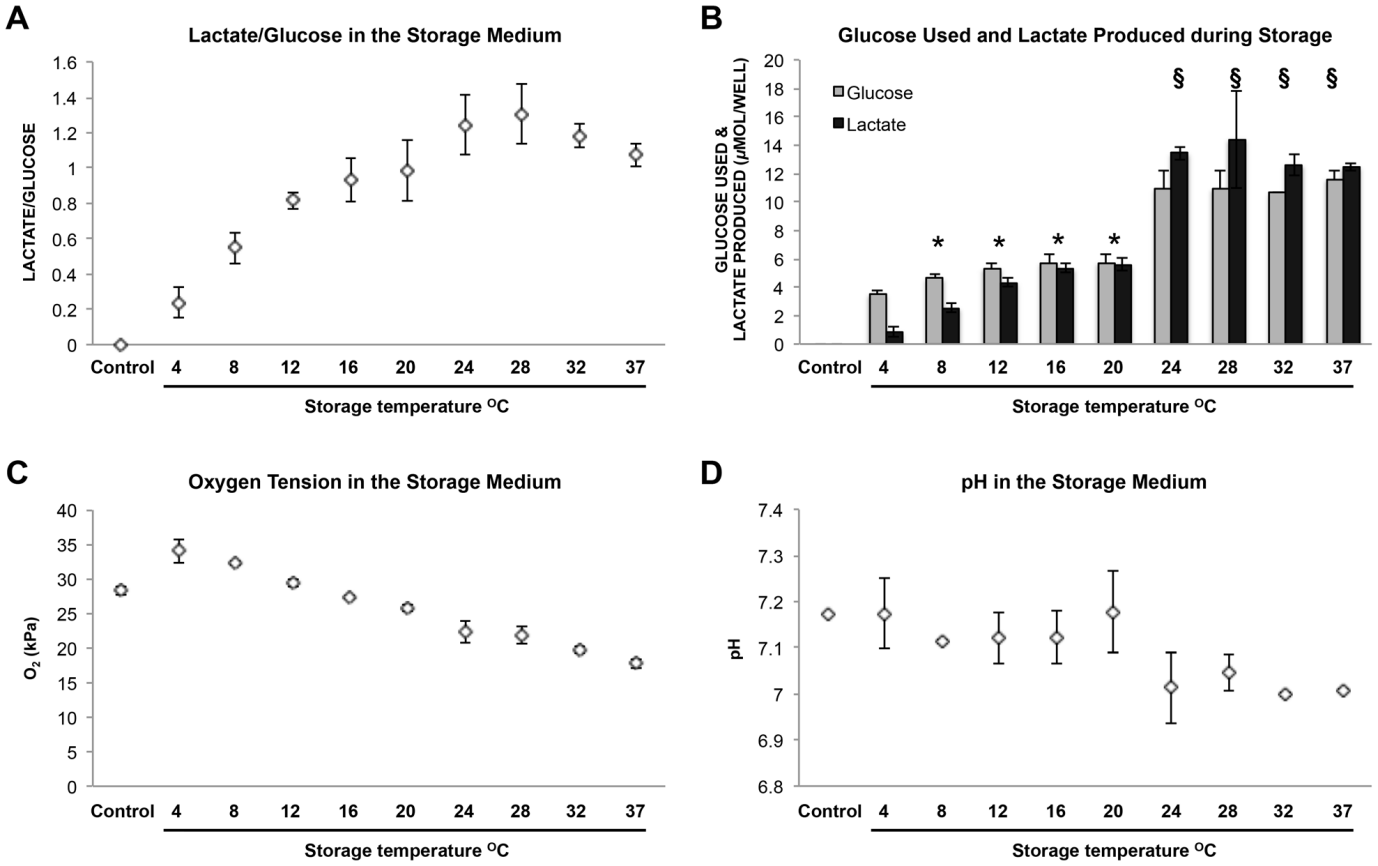
Metabolic measurements taken from the cell viability experiment after two weeks storage. (A) Lactate/glucose values increased with temperature (*r*  =  0.927; *p* < 0.001 between 4°C and 28°C). (B) Glucose and lactate values were significantly grouped between 8°C and 20°C (* =  significantly grouped - (glucose: *p* > 0.05; lactate: *p* > 0.05)) and values were approximately double between 24°C and 37°C (§  =  significantly grouped - (glucose: *p* > 0.05; lactate: *p* > 0.05)). (C) Oxygen tension values were inversely correlated with temperature (*r*  =  - 0.939; *p* <0.001). (D) pH fluctuated between pH 7.1 and pH 7.2 from 4°C to 20°C. Decreased pH fluctuating around pH 7.0 reflected higher lactate production between 24°C and 37°C. Values represent the average seen in n  =  4 wells of a 12 well plate at each temperature.

The high lactate/glucose (L/G) ratios found at higher temperatures suggested that the glycolytic pathway accounted for a large part of energy production from glucose (at the highest, approximately 66% of glucose used was converted to lactate, given that the maximum possible L/G ratio is 2) (Figure 3A). The strongest correlation between the L/G ratio and temperature was seen between 4°C and 28°C (*r* = 0.927; *p*<0.001). L/G ratio was lowest in the 4°C group (0.25±0.08), and the peak L/G value was seen after storage at 28°C (1.31±0.17). A lower L/G ratio relative to 28°C was seen at 32°C and 37°C (1.07±0.06) (Figure 3A).

Storage temperature effect on metabolic values could be bracketed into two distinct groups. The first group occurred between 8°C and 20°C where average glucose use was 5.36±0.42 *µ*mol/well and average lactate production was 4.45±0.35 *µ*mol/well (significantly similarly grouped (glucose: *p*>0.05; lactate: *p*>0.05)) (Figure 3B). A marked increase in metabolic activity was seen between 20°C and 24°C. The second group occurred between 24°C and 37°C, where both glucose use and lactate production were over twice that seen at lower temperatures; average glucose use was 11.03±0.80 *µ*mol/well and average lactate production was 13.23±1.22 *µ*mol/well (significantly similarly grouped (glucose: *p*>0.05; lactate: *p*>0.05)). Thus the highest glucose use and lactate production were seen at higher temperatures (Table S1, Figure 3B).

PO_2_ values correlated inversely with increased temperature (*r* = −0.939; *p*<0.001), starting at 34.05 kPa (34% PO_2_) at 4°C and decreasing steadily to 17.75 kPa (17.5% PO_2_) at 37°C (Figure 3C). The control value was 28.35 KPa (28% PO_2_), measured in the medium alone. The average in cell culture medium (KM) taken from cell cultures before storage was 22.75 kPa (22% PO_2_). PO_2_ was also inversely correlated with L/G ratio (*r* = −0.808; *p*<0.010) (data not shown).

KM in cell cultures before storage had an average pH of 7.47. During storage, small fluctuations ranging between pH 7.1 and pH 7.2 were seen from 4°C to 20°C and fluctuations around pH 7.0 were seen between 24°C and 37°C, reflecting the increase in lactate production at higher temperatures (Figure 3D).

### Assessment of Metabolic Values in Volumes Used in Other Experimental Groups

**Figure 4:**
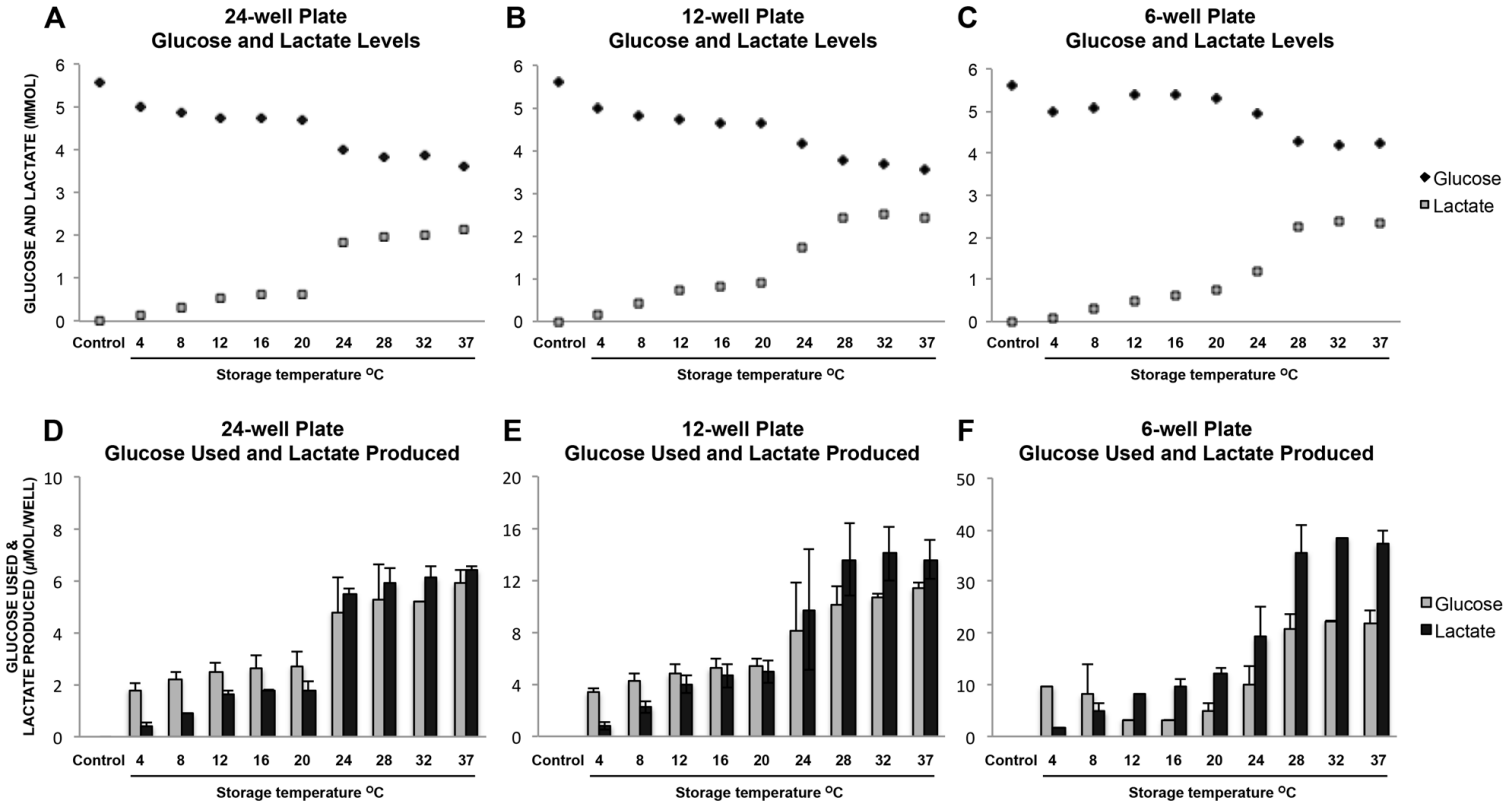
Metabolic measurements after two weeks storage showing different volumes. (A) (B) (C) Glucose and lactate concentration. (D) (E) (F) Glucose used and lactate produced (calculated from (A) (B) (C)). (A) and (D) 24 well plate volume 2mL, area 1.9 cm^2^ (n  =  3). (B) and (E) 12 well plate volume 5.6mL, area 3.5 cm^2^ (n  =  7). (C) and (F) 6 well plate volume 16 mL, area 9.6 cm^2^ (n  =  3).

The profile of glucose use and lactate production throughout the temperature range in other experimental volumes (24-well and 6-well) largely reflected the results described above in the cell viability experiment (12-well) (Figure 4A – F). Of note, no variation in metabolic values corresponding to storage volume was revealed.

### Cell Morphology

**Figure 5:**
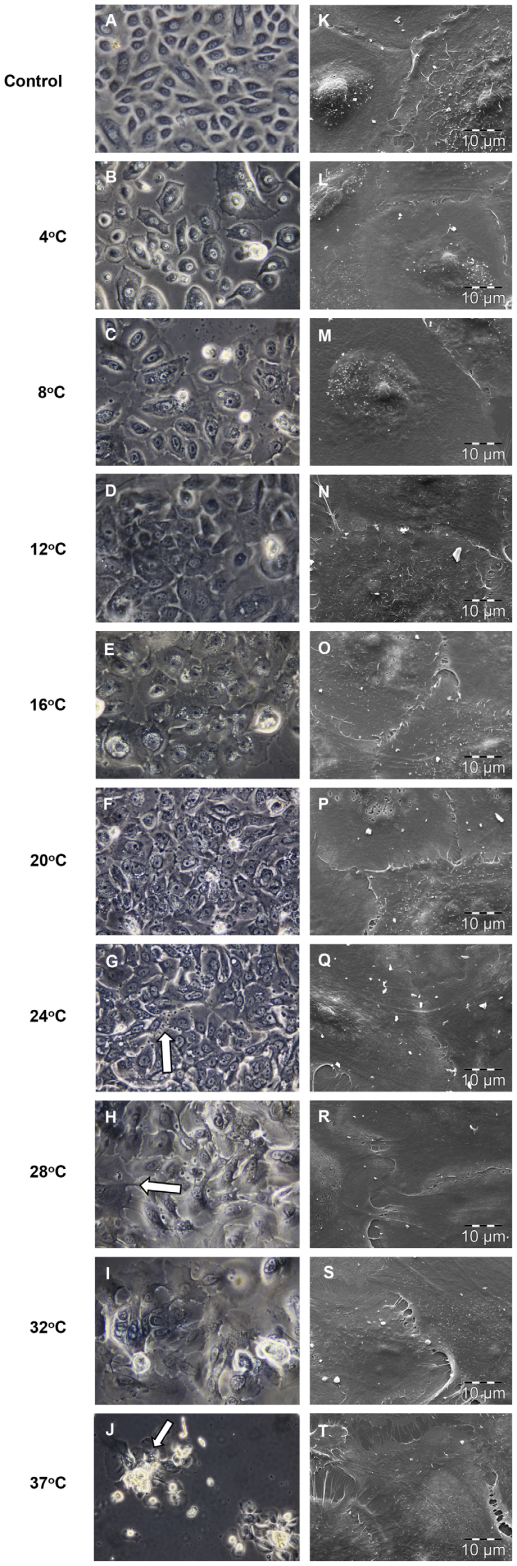
Morphology shown by phase contrast (X400) and SEM (X2000) micrographs. Morphology was most similar to non-stored control at 12°C and 16°C ((D) (E) (N) and (O)). Cell shrinkage was apparent at 4°C and 8°C ((B) and (C)); granulation (arrow (G)) and flattened cell morphology (arrow (H)) suggested more differentiation at higher temperatures ((F) - (J)). Cell clusters were seen at higher temperatures (arrow (J)). Representative SEM micrographs show preservation of cell-cell contact in selected areas ((L) – (T)) (n  =  3).

HEKa cells were grown to confluence and had a characteristic cobblestone appearance before storage. Phase contrast micrographs were taken after exchanging storage media for KM and a 3 hour adjustment period in the 37°C incubator. Morphology most similar to control was observed at 12°C and 16°C, where cell-cell contact within epithelial sheets was well maintained (Figure 5A, D, and E). The presence of elongated filopodia, and an enlarged flattened appearance suggested more differentiation at temperatures above 16°C. In addition, dark granules were seen at the plasma membrane in some cells at 20°C and 24°C (Figure 5F and G). Changes in epithelial sheet morphology were also seen at temperatures above 16°C, with less distinct separation between adjacent cells. There was a tendency for cells to stream together at 28°C, 32°C and 37°C (Figure 5H - J). Clustered cells formed uneven islands at 32°C and 37°C (Figure 5H - J). Abnormally large intercellular space, indicative of cell shrinkage, was seen at 4°C and 8°C (Figure 5B).

Scanning electron microscopy (magnification: X2000) showed that cell-cell contact was similar to that of control cells at most temperatures in selected confluent areas (Figure 5K – T). Retention of individual cells with microvilli on the apical surface similar to the non-stored control was seen at all temperatures between 4°C and 24°C (Figure 5L – O).

### Phenotype Analysis by Immunocytochemistry

**Figure 6:**
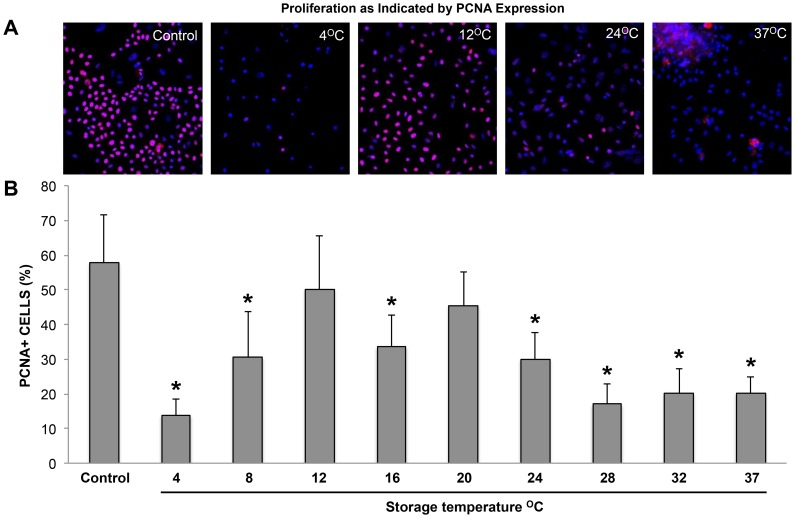
Proliferation as indicated by PCNA positive cells. (A) Representative images of PCNA staining (TRITC - red) combined with nuclear staining (DAPI – blue) showing 4°C, 12°C, 24°C and 37°C compared to non-stored control. Expression was significantly reduced at all temperatures (*  =  significantly reduced, *p* < 0.05) except at 12°C and 20°C (B); significantly higher PCNA expression was seen at 12°C with 50 ± 15% and 20°C with 46 ± 10%, where values were comparable to the control value of 57 ± 14% (*p*  =  0.983 and 0.752, respectively).

The percentage of Proliferating Cell Nuclear Antigen (PCNA) positive cells is presented in graphical form to show comparison of expression between temperatures (Figure 6B). The percentage shown at 32°C and 37°C is an estimate, as counts were difficult to determine accurately due to clustering of cells. Since cell viability between temperatures varied significantly, and some dead cells were detached and lost during media change and fixation, it should be noted that values indicate the average percentage of remaining cells positive for the marker (n = 4). In control, PCNA was confined mostly to smaller cells that showed a very strong nuclear expression indicative of active proliferation (Figure 6A) [25]. Lowest expression was seen after storage at 4°C (Figure 6B). Expression was significantly reduced at all temperatures (*p*<0.05) except at 12°C (Figure 6B) and 20°C; significantly higher expression was seen at 12°C with 50±15% and 20°C with 46±10%, where values were comparable to the control value of 57±14% (*p* = 0.983 and 0.752, respectively). Fluorescence intensity began to weaken at 24°C (Figure 6A), and significantly lower expression compared to control was seen from 24°C to 37°C, at approximately 25% (*p*<0.05). Weak expression, confined to the center of aggregated cell clusters was characteristic of cells stored between 28°C and 37°C (Figure 6A).

## Discussion

In the present study, the morphology, cell viability, phenotype and metabolic status following two weeks of xenobiotic-free storage of CECS were assessed. Glucose and lactate values in the storage media gave an indication of metabolic status and provided further evaluation of cell viability [26], [27], whereas oxygen tension and pH measurements ensured storage conditions were within a physiologically relevant range.

Low cell viability values seen at 4°C and 8°C were corroborated by a high proportion of dead cells and low glucose consumption at these temperatures. In addition, phase contrast microscopy showed cell-cell contact was disrupted and cell shrinkage was apparent at 4°C and 8°C, indicative of apoptosis [28]. Retention of proliferative function was lowest at 4°C also confirming poor cell survival at this temperature. Low cell viability (∼20%) seen at 4°C is in agreement with other studies that have shown cell viability ranging from ∼20% to ∼33% after storage at this temperature [5], [12]. Rewarming after cold-storage has been shown to induce mitochondrial swelling and initiate apoptosis, which may account for the high cell death seen at 4°C [23], [29]. Evidence of injury to epidermal cells associated with cold and re-warming seen at 4°C is particularly clinically relevant as refrigeration is the most common method of split skin graft storage and low cell viability may influence skin graft healing [5].

High cell viability at 24°C (∼95%) shown here after 14 days of storage is contrary to that demonstrated in a study of pig skin at 25°C where cell viability declined to approximately 60% within one day [11]. Different storage conditions, inherent differences in cell function found between intact skin tissue and CECS, and species differences may explain the different results. Comparable cell viability has been demonstrated after storage of cultured limbal and conjunctival epithelial cells at 23°C for seven days [30], [31]. A smaller peak in cell viability was seen at 12°C (∼60%) which coincided with highest PCNA expression. A temperature study on storage of lung endothelial cells also reported similar results at 10°C [23]. The favorable conditions at 10°C were explained by minimal generation of catalytically available intracellular iron and reduced oxidative stress. Similarly, high proliferative capability was retained [23]. Higher cell viability, at approximately 60% or above, was shown in the present study at several temperatures (12°C, 24°C, 28°C, 32°C and 37°C), which is comparable to that reported in cultured epithelial autograft cryopreservation studies [27], [32]. When combining PCNA results with cell viability, 12°C and 24°C appeared to be the most promising in terms of conservation of viable, proliferating cells following storage.

In accordance with other studies [33], cell viability as detected by CAM fluorescence was strongly correlated with glucose use. Exceptions were seen at 12°C and 24°C, where cell viability was higher, suggesting that conditions were especially favorable to efficient energy use. Optimum oxygen tension for keratinocyte growth has been shown at 18% O_2_ [34] and lower (2% O_2_) [35]. Therefore, the dampening effect of high oxygen tension may have contributed to the low cell viability found at 4°C (34% O_2_), and the increase in cell viability and metabolic values seen between 24°C (22% O_2_) and 37°C (17.5% O_2_). In agreement with van't Hoff's principle stating that metabolic activity decreases by a factor of 1.5-2X for each 10°C drop in temperature, metabolic values (glucose use and lactate produced) at 28°C were on average twice that seen at 20°C [36]. However, the difference in metabolic activity between high and low temperatures did not decline in a continuous fashion; a sharp drop was seen below 24°C, accompanied by a notable decrease in the L/G ratio. From 20°C to 8°C, the decline in glucose use was more moderate, suggesting that epidermal cells may have an inbuilt adaptation to cooler temperatures below 24°C.

Lactate production was seen at all temperatures, despite sufficient oxygen to facilitate oxidative phosphorylation. This suggests that, at least in part, metabolic function was fulfilled through the use of the less efficient glycolytic pathway under aerobic conditions. The importance of glycolytic metabolism was particularly emphasized at high temperatures where L/G ratios were higher. In a comparable study, incubation of epidermal biopsies with or without oxygen resulted in no significant difference in lactate production [37]. Aerobic glycolysis, also known as the Warburg effect, has been described in cells with high energy requirements and proliferation rates [38], and it has been well documented that skin converts a high percentage of glucose to lactate [39]. Furthermore, oxidative phosphorylation has been found to be particularly dispensable in epidermal stem cells in favor of aerobic glycolysis [40]. Preference for use of the glycolytic pathway instead of oxidative phosphorylation may represent a defense mechanism against reactive oxygen species (ROS) generation, side-products of normal mitochondrial aerobic phosphorylation in situations of high-energy demand [41], [42]. Thus the lactate production seen here confirms other findings showing the reliance of epidermal cells on glycolysis [37], [39]. The distinct difference in metabolic values at high, compared to low, temperatures suggests a possible advantage of storage at temperatures below 20°C, due to a slower metabolic rate.

High L/G ratios were consistently seen at temperatures above 20°C regardless of storage volume. As the present study is the first to investigate the effect of storage temperature on the L/G ratio in CECS, direct comparisons were not possible. However, a high L/G ratio in CECS after 16 hours in a 37°C incubator following cryopreservation has been shown [9].This corroborates results shown here suggesting that glycolysis plays a fundamental role in serving energy needs even under aerobic conditions, especially at higher temperatures approximating normal skin temperature of 30°C [43], where normal metabolic rate may be expected.

Cell-cell and cell sheet architecture most resembling that of non-stored control cells was seen at 12°C and 16°C, whereas storage at temperatures above 20°C appeared to induce more differentiation [44]. Epidermal sheet disruption and rounded, uneven cell clusters, reminiscent of those seen in the disorganized epidermis of ΔNp63α-null mice were characteristic morphological features at 32°C and 37°C [45].

The number of viable cells remaining at the end of the storage period is a natural product of cell proliferation and cell turnover, therefore the contribution of cell proliferation to cell viability is of particular interest. In addition, continued proliferative function is especially relevant to cell sheet integration after surgery. PCNA staining varied widely throughout the temperature range indicating that storage temperature had a profound effect on proliferation. Cell viability was positively correlated with PCNA expression and trends below and above the best fit line between these two assessments indicated that proliferation played a more significant role in preservation of a viable cell population at lower temperatures, particularly at 12°C and 20°C. Conversely, weaker and lower PCNA expression was consistently seen at 24°C and temperatures above, despite generally higher cell viability and metabolic values. PCNA expression was mostly localized to smaller cells at every temperature. As small cell size is associated with stem cell character [46], increased differentiation at higher temperatures as indicated by increased occurrence of larger spreading cells and other morphological features, may have been responsible for lower PCNA expression.

The present study illustrates that temperature can have a profound effect on cell viability, morphology, and phenotype of CECS during storage. However, the use of cells from a single donor may be considered limiting. Factors that may affect extrapolation of these results include variation in donor cells as a consequence of systemic factors, such as donor health [47], [48] and age [49]. Of note, studies comparing cell culture growth between donors have shown that the procurement and handling of starting material [47], [49], [50], as well as the health status of the individual donor [47], [48], have a greater impact on cell growth when compared to the effect of age, supporting the use of cells from a young healthy donor in this study.

In conclusion, combined results suggest that the most extreme storage temperatures (4–8°C and 32–37°C) should be avoided for storage of CECS. Given the clear advantage of 24°C on cell viability, this may be the best temperature choice for the storage of cultured epidermal cells in preparation for surgery. Improved stability due to differentiation associated changes may have contributed to higher cell viability at this temperature. However, results at 12°C were particularly favorable in terms of maintenance of proliferative capacity and morphology of cultured cells after storage, while providing more than 60% cell viability. Metabolic values at 12°C suggest metabolism was dampened yet sufficient to allow slow cell growth, sustain intracellular homeostasis and a viable cell sheet population. Collectively, the data suggest that both 12°C and 24°C are strong candidates for storage of cultured epidermal cells that merit further investigation.

## Supporting Information

Table S1 **Values represent the average of n = 4 replicates for each temperature stored in a 12-well plate.** Control values are those present in the storage medium on day 1.
(DOCX)
