# Supplementary material for: Effect of Storage Temperature on Cultured Epidermal Cell Sheets Stored in Xenobiotic-Free Medium
Source: PLoS One. 2014 Aug 29;9(8):e105808. doi: 10.1371/journal.pone.0105808 (PMC4149437; doi:10.1371/journal.pone.0105808)
Supplement: Table S1 — Values represent the average of n = 4 replicates for each temperature stored in a 12-well plate. Control values are those present in the storage medium on day 1. (DOCX) [file pone.0105808.s001.docx]

|  | **Average Glucose, Lactate and Lactate/Glucose (L/G) Values** | | | | | | | | | |  |  |  |  |
| --- | --- | --- | --- | --- | --- | --- | --- | --- | --- | --- | --- | --- | --- | --- |
| **Temperature (^0^C)** | **Glucose Measured (mMol/L)** | **SD** | **Glucose used (uMol/well)** | **SD** | **Lactate measured (mMol/L)** | **SD** | **Lactate produced (uMol/well)** | **SD** | **Avg L/G** | **SD** | **pO2 (kPa)** | **SD** | **pH** | **SD** |
| Avg in culture before switch to storage medium (n=7) | 5.04 | 0.32 | # | # | 1.47 | 0.44 | # | # | 0.30 | 0.11 | 22.75 | 0.41 | 7.47 | 0.05 |
| Control – Day 1 storage medium | 5.60 | 0.00 | 0.00 | 0.00 | 0.00 | 0.00 | 0.00 | 0.00 | 0.00 | 0.00 | 28.35 | 0.64 | 7.17 | 0.00 |
| 4 | 4.98 | 0.05 | 3.50 | 0.28 | 0.15 | 0.06 | 0.84 | 0.32 | 0.24 | 0.08 | 34.05 | 1.70 | 7.17 | 0.08 |
| 8 | 4.78 | 0.05 | 4.62 | 0.28 | 0.45 | 0.06 | 2.52 | 0.32 | 0.55 | 0.09 | 32.35 | 0.35 | 7.11 | 0.00 |
| 12 | 4.65 | 0.06 | 5.32 | 0.32 | 0.78 | 0.05 | 4.34 | 0.28 | 0.82 | 0.05 | 29.40 | 0.52 | 7.12 | 0.06 |
| 16 | 4.58 | 0.10 | 5.74 | 0.54 | 0.95 | 0.06 | 5.32 | 0.32 | 0.93 | 0.12 | 27.38 | 0.21 | 7.12 | 0.06 |
| 20 | 4.58 | 0.10 | 5.74 | 0.54 | 1.00 | 0.08 | 5.60 | 0.46 | 0.99 | 0.17 | 25.78 | 0.44 | 7.18 | 0.09 |
| 24 | 3.65 | 0.24 | 10.92 | 1.33 | 2.40 | 0.08 | 13.44 | 0.46 | 1.25 | 0.17 | 22.33 | 1.60 | 7.01 | 0.08 |
| 28 | 3.65 | 0.24 | 10.92 | 1.33 | 2.58 | 0.61 | 14.42 | 3.43 | 1.31 | 0.17 | 21.93 | 1.30 | 7.05 | 0.04 |
| 32 | 3.70 | 0.00 | 10.64 | 0.00 | 2.25 | 0.13 | 12.60 | 0.72 | 1.18 | 0.07 | 19.70 | 0.41 | 7.00 | 0.00 |
| 37 | 3.53 | 0.10 | 11.62 | 0.54 | 2.23 | 0.05 | 12.46 | 0.28 | 1.07 | 0.06 | 17.75 | 0.61 | 7.01 | 0.00 |

Table S1: Supplementary Table S1
